# Isolated non-immune hydrops fetalis: an observational study on complete spontaneous resolution, perinatal outcome, and long-term follow-up

**DOI:** 10.1007/s00404-022-06731-w

**Published:** 2022-08-22

**Authors:** Sophie Neveling, Alexander Johannes Knippel, Peter Kozlowski

**Affiliations:** 1 Heinrich-Heine-University, Duesseldorf, Germany; 2 Praenatal-Medizin und Genetik, Duesseldorf, Germany

**Keywords:** Fetal diseases, Functional impairment, Isolated non-immune hydrops fetalis, Long-term follow-up, NIHF, Spontaneous resolution

## Abstract

**Purpose:**

The aim of our study was to investigate spontaneous resolution and postnatal outcome in non-immune hydrops fetalis (NIHF). We specifically studied NIHF cases that occurred without any other anomalies in the prenatal diagnostic workup, defined as isolated NIHF (iNIHF).

**Methods:**

To identify iNIHF we retrospectively classified prenatal findings of 700 NIHF singletons, diagnosed in our prenatal referral center between 1997 and 2016. We studied the occurrence of prenatal resolution in iNIHF and linked it to the perinatal outcome. We obtained long-term outcome by contacting the parents, children, and the pediatricians and listed all functional and structural anomalies and temporary logopedic, psychosocial and motoric impairments.

**Results:**

Among 70 iNIHF cases, 54 (77.1%) resolved completely prenatally. The baby-take-home rate was 98.1% in these cases. In contrast, the baby-take-home rate in the subgroup without complete resolution was 25.0%.

We achieved pediatric long-term outcome in 27 of 57 survivors (47.4%) of iNIHF with a mean follow-up period of 10.9 years. Among these 27 children, fetal hydrops had completely resolved prenatally in 26 cases and had regressed to a mild effusion in one case. In the pediatric development, two children had significant functional impairment and two children showed recurrent skin edema.

**Conclusion:**

Complete spontaneous resolution was the most common intrauterine course of iNIHF in our collective. Completely resolved iNIHF had a favorable perinatal outcome in our study. Our data on the long-term outcomes are consistent with the assumption of an increased rate of functional impairments.

**Trial registry:**

Internal study number of Heinrich-Heine-University, Duesseldorf: 6177R. Date of registration: December 2017.

**Supplementary Information:**

The online version contains supplementary material available at 10.1007/s00404-022-06731-w.

## What does this study add to the clinical work

**Table:** 

This study provides comprehensive outcome data from a cohort of isolated non-immune hydrops fetalis (iNIHF) in which the most common prenatal course was spontaneous resolution. The data may be helpful in counseling expectant parents about survival chances as well as the spectrum of pediatric anomalies and functional impairments.	

## Introduction

Non-immune hydrops fetalis (NIHF) is described as a dynamic process of pathological fluid accumulations in fetal compartments that is unrelated to red cell alloimmunization [1, 2].

For the etiological classification of NIHF, systematic reviews have grouped fetal anomalies associated with NIHF into diagnostic categories [1, 3, 4].

In recent studies that evaluate whole exome sequencing (WES) to identify the cause of NIHF, the categorization is based on the linkage of phenotypic and genetic findings using constantly adapting databases [2, 5–9].

Nevertheless, the etiology of NIHF remains unknown in several cases, and most studies classify these conditions as idiopathic hydrops fetalis [3, 4, 10–15].

Hydropic fetuses with no further pathologies in high-resolution ultrasound, and with normal results in immuno- and infection diagnostics, as well as in cause-related molecular genetic tests (e.g., in suspected metabolic diseases) are still of considerable clinical relevance. To focus on the regular prenatal manifestation of this condition, we studied isolated non-immune hydrops fetalis (iNIHF) as a subgroup from the former idiopathic group. This group consists only of NIHF cases that underwent a complete diagnostic workup and had unremarkable findings apart from hydrops fetalis.

In general, the outcomes of NIHF are poor and depend on the etiology [1, 11]. For euploid NIHF without fetal structural anomalies Hartge et al. describe favorable survival rates [14]. To further investigate this observation, we specifically investigated the survival and long-term outcome of iNIHF.

Furthermore, a positive correlation between prenatal resolution of hydrops fetalis and the survival outcome has been reported [11, 16]. Spontaneous resolution is mostly described in pregnancies with infections and well researched in transplacental parvovirus B19 infections [17, 18]. Recently there were reports of fetal transient skin edema in three pregnancies affected by COVID-19 [19, 20]. Concerning non-infectious euploid NIHF cases without fetal structural anomalies, there are only few case reports on spontaneous resolution [21–26].

Our aim was to investigate prenatal resolution in a large series of iNIHF and to follow-up the postnatal and long-term outcome.

To date, only a few studies have assessed the pediatric development after NIHF for more than one year [27–30]. Follow-up data on long-term morbidity and quality of life assessments are lacking [11]. We followed-up the pediatric development after iNIHF for all ages to identify functional limitations and to enable conclusions to be drawn from anomalies to the pathogenesis of hydrops fetalis. Bellini and Boudon et al. suppose that dysplasia of the lymphatic vessels could be a cause of unexplained hydrops fetalis [31, 32]. Thus, we specifically searched for prenatally undiagnosed lymphatic dysfunctions in the pediatric reports of the long-term development after iNIHF.

## Methods

**Fig. 1:**
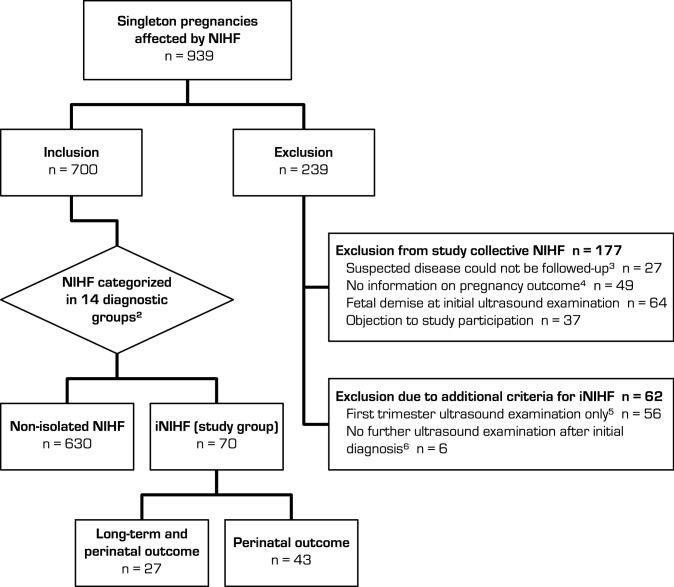
Generation of the study group ‘isolated non-immune hydrops fetalis’ (iNIHF)^1^**.** iNIHF: isolated non-immune hydrops fetalis; NIHF: non-immune hydrops fetalis.^1^ Nonimmune hydrops fetalis without any malformations, or other clinically relevant anomalies in prenatal diagnostic workup. ^2^Diagnostic groups based on the definition by Bellini et al. [3]. ^3^Lack of clarifying diagnostics due to fetal demise (*n* = 8) or objection to further diagnostics (*n* = 19). ^4^Neither reports on postnatal follow-up until discharge from hospital nor prenatally confirmed positive laboratory diagnosis. ^5^Fetal demises before second trimester (*n* = 52), no ultrasound examination after first trimester in our center (*n* = 4). ^6^Exclusion due to missing information concerning the development of NIHF

This retrospective observational study was conducted at a prenatal tertiary referral center, investigating a cohort of all singleton pregnancies with a diagnosis of NIHF between April 1997 and March 2016 (*n* = 939, see Fig. 1).

For prenatal data, a query was performed in our database (ViewPoint^©^, GE Healthcare GmbH, Germany), combining all information on our ultrasound results, laboratory findings, and medical history, as well as external examination records of the referring gynecologist.

We searched for reports containing the term ‘hydrops’ in any result text of an ultrasound scan in the course of pregnancy and reviewed all examinations. We defined cases as ‘hydrops fetalis’ if in at least one of our sonographic scans, two or more of the following pathological fetal fluid accumulations were present: pleural effusion, pericardial effusion, ascites or anasarca. We based our definition of NIHF on the current clinical guideline by the Society for Maternal–Fetal Medicine [1].

### Classification of NIHF

We categorized all included fetuses in 14 diagnostic groups based on the etiological classification of NIHF by Bellini et al. [3]. Differing from this classification, we created the group of isolated NIHF (iNIHF) as a subgroup of the former idiopathic NIHF category. We allocated hydropic fetuses to the iNIHF group, if no further clinically relevant anomaly in addition to hydrops fetalis was found in the complete prenatal diagnostic workup as described below.

Furthermore, we classified all non-isolated NIHFs into the remaining 13 diagnostic groups: chromosomal, cardiovascular, infectious, syndromes, thoracic, urinary tract malformations, lymphatic, feto-maternal, gastrointestinal, metabolic, extra thoracic tumors, hematologic, and miscellaneous.

For the classification of fetuses with multiple anomalies, genetic diseases, infections, and metabolic diseases, each confirmed by laboratory findings, were prioritized over ultrasound results. If multiple sonographic anomalies were present, we prioritized the anomaly most frequently reported to be in correlation with hydrops fetalis over the other sonographic findings [1]. If phenotypical clusters were typical for a non-chromosomal syndrome, they were classified into the syndromic group.

### Diagnostic workup, inclusion, and exclusion criteria

For the inclusion and etiological classification of NIHF cases (see Fig. 1) all the following diagnostic criteria had to be met: ultrasound examinations, echocardiography and doppler examinations had to be performed in our center by at least DEGUM (German Society of Ultrasound in Medicine) level II certified specialists in obstetric ultrasound using high resolution equipment. We required the availability of postnatal outcome reports. If prenatal laboratory reports of genetic and infectious diseases based on fetal samples were positive, we considered them as reliable as perinatal outcome reports.

Additional diagnostic requirements for the inclusion into the study group of isolated NIHF were the procedure of more than one ultrasound examination in our center, at least one of them performed after the first trimester, and a result of normal infectious screening from maternal serum and normal karyotype after invasive diagnostics. Balanced Robertsonian translocation and triple X were considered non-pathological.

### Resolution of iNIHF

Spontaneous resolution was defined as complete resolution of skin edema and effusions in any body cavity that did not reoccur in the course of pregnancy and was not induced by therapeutical intervention. Additionally, we termed fluid accumulations in either one single body cavity or exclusively present skin edema (including nuchal edema) as single compartment effusion.

### Perinatal and long-term follow-up of iNIHF

Information on perinatal outcome was obtained from standardized questionnaires that all parents were asked to return. In non-responders the information was requested by phone call or, if consent of the patient was obtained, from the referring physician.

To acquire long-term outcome, we contacted all families with survivors of iNIHF via phone calls. Moreover, information letters on the study were sent to the parents and additionally age-appropriate versions to children from the age of six.

If informed consent was given, pediatricians were contacted for retrospective data on the development and current health. Results of routine examinations (‘U1–11’ according to German pediatric guidelines, GBA), further specialist examinations and clinical reports were collected. Simultaneously, parents were asked for their perception of the child's health status. We defined major congenital disorders as resulting in permanent severe functional impairment. Minor anomalies were defined as congenital disorders leading to permanent or temporary mild functional impairment. Temporary logopedic, psychosocial and motoric anomalies in which parents reported inconspicuous overall development were listed separately.

The collection of long-term data was conducted in 2018. The study design was approved by the ethics committee of Heinrich-Heine-University, Duesseldorf (internal study number 6177R).

## Results

A total of 201,351 singleton pregnancies was sonographically examined in our center during the study period. Among them 939 fetuses (0.47%) met the diagnostic criteria for NIHF, of which 700 cases (74.5%) could be included in our study. Isolated NIHF was diagnosed in 70 of all included fetuses (10.0%). The other 630 fetuses (90.0%) had a diagnosis of at least one anomaly and were classified as non-isolated NIHF. Characteristics of the isolated and non-isolated group are shown in Table 1.

**Table 1:** Characteristics of included cases

	Mean GA at first diagnosis (w)	Mean maternal age at first diagnosis (y)	Mean maternal BMI	No
iNIHF	12.93	30.62	23.58	70
Non-isolated NIHF	14.62	32.96	24.24	630
Total	14.45	32.72	24.16	700

*BMI* Body mass index (kilogram/square meter), *GA* Gestational age, *iNIHF* isolated non-immune hydrops fetalis, *NIHF* non-immune hydrops fetalis, *w* weeks, *y* years

### Characteristics and outcome of iNIHF

We observed complete spontaneous resolution of hydrops fetalis within pregnancy in 54 of 70 iNIHF cases (77.1%). Prenatal characteristics differed between the groups with and without resolution of hydrops in the mean gestational age (GA) at initial diagnosis (mean GA 11.50 weeks and 17.78 weeks) and mean GA at initial examination (mean GA 11.41 weeks and 15.63 weeks, Table 2). Besides that, we did not find any relevant differences in the prenatal characteristics (maternal age, body-mass-index) of these groups.

**Table 2:** Spontaneous resolution of iNIHF within pregnancy – Baby-take home rate and GA at first examination, first diagnosis and resolution of hf

	Total no (%)	Demise^a^ no (%)	Baby-take- home no (%)	Mean GA at first ultrasound examination	Mean GA at first diagnosis of hf	Mean GA at complete resolution of hf	Mean interval^b^	Mean GA at birth/demise
**No complete resolution**	**16 (22.9%)**	**12 (92.3%)**	**4 (7.0%)**	**15.63**	**17.78**		**7.70**	**25.48**
**Complete resolution**	**54 (77.1%)**	**1 (7.7%)**	**53 (93.0%)**	**11.41**	**11.50**	**17.79**	**6.29**	**39.01**
In first trimester	12 (17.1%)		12 (21.1%)	10.60	10.77	12.45	1.68	39.24
In second trimester	40 (57.1%)	1 (7.7%)	39 (68.4%)	11.62	11.65	18.58	6.93	38.88
In third trimester	2 (2.9%)		2 (3.5%)	12.14	12.86	34.00	21.14	40.07
**Total**	**70 (100%)**	**13 (100%)**	**57 (100%)**	**12.37**	**12.93**	**17.79**	**6.61**	**35.91**

*GA* Gestational age (in weeks), *hf* Hydrops fetalis, *iNIHF* isolated non-immune hydrops fetalis

First trimester of pregnancy: GA < 14 + 0 weeks; second trimester: GA 14 + 0–27 + 6 weeks; third trimester: GA > 28 + 0 weeks

^a^Demise included termination of pregnancy (*n* = 6), spontaneous abortion (*n* = 3) and postnatal death (*n* = 4)

^b^Mean interval between first diagnosis and complete resolution of hydrops fetalis/birth/demise (in weeks)

In total, the baby-take-home rate was 81.4%, 57 of the 70 fetuses with iNIHF survived (For details see Table 2).

In the subgroup with complete resolution of hydrops fetalis, the baby-take-home rate was 98.1% (53 of 54), one preterm delivery with neonatal demise occurred at 27 + 1 weeks. In this case first diagnosis was at 11 + 1 weeks and complete resolution was at 19 + 6 weeks. The fetus showed an increased nuchal translucency of 6 mm at 11 + 1 weeks and a slight dilatation of an intestinal loop at 19 + 6 weeks.

In contrast, the baby-take-home rate was 25.0% (4 of 16) in the subgroup without complete resolution. The 12 demises included 2 cases of spontaneous fetal loss, 4 cases of postnatal death within a week after birth and 6 cases of termination of pregnancy (TOP) e.g., due to the massive progression of hydrops fetalis.

Results of the long-term follow-up could be obtained in 27 of 57 survivors (47.4%). Complete resolution of hydrops fetalis had prenatally occurred in 26 of these children and had regressed up to a mild effusion in one case. The mean follow-up period was 10.9 years (range 2–18). In the other 30 (52.6%) surviving children, obstetric data up to discharge of the hospital (U1/2) was available. All details and an individual case survey are described in Table 3.

**Table 3:** Individual case survey on anomalies in the long-term development of iNIHF

	Anomalies	Follow-up (years)	Prenatal resolution of NIHF	Non-temporary anomalies	Temporary anomalies and additional mild logopedic, psychosocial and motoric impairment^a^	Birth at GA (completed weeks + days)
1	Major anomaly	14	Yes	**Autism spectrum disorder, ataxia**	Psychosocial anomalies: aggressive social behavior disorder (U9)	38 + 3
2	Major anomaly	13	Yes	**Severely disabled, global developmental delay, autism, facial stigmata** Variants with unclear clinical relevance were found in aCGH analysis^b^		38 + 5
3	Minor anomaly	18	Yes	Neurodermatitis, bronchial asthma	Logopedic anomalies: sigmatism, dyslalia, mild hearing impairment (U8)	39 + 5
4	Minor anomaly	16	Yes	Congenital hip dysplasia, joint pain, Raynaud's disease		40 + 0
5	Minor anomaly	12	Yes	Neurodermatitis	Cryptorchidism (U7) Logopedic anomalies: sigmatism (U9) Psychosocial anomalies: encopresis (U10)	40 + 1
6	Minor anomaly	11	Yes	Abnormal posture of a kidney	Umbilical hernia (U7) Logopedic anomalies: dyslalia (U8) Psychosocial anomalies: social anxiety (U8), nocturnal enuresis (U9)	38 + 6
7	Minor anomaly	11	Yes	Selective IGA deficiency		39 + 5
8	Minor anomaly	11	Yes	**Recurrent hand and feet edema** Variants with unclear clinical relevance were found in aCGH analysis^c^	Suspected syndrome (U1–7) due to combination of hypospadias, cryptorchidism, hemangioma, hypertelorism, examinations of metabolism inconspicuous, overall normal development and growth	39 + 3
9	Minor anomaly	9	Yes		Sinus pilonidal (U2–3) Logopedic anomalies: sigmatism (U8)	40 + 0
10	Minor anomaly	9	Yes	Congenital hip dysplasia	Logopedic anomalies: dyslalia (U8) Motoric anomalies: abnormal fine motor skills (U9)	39 + 2
11	Minor anomaly	8	Yes		Small palatal cyst (U2–6) Logopedic anomalies: dyslalia (U8) Psychosocial development anomalies (U9)	38 + 6
12	Minor anomaly	6	Yes	**Recurrent feet edema**		39 + 6
13	Minor anomaly	6	No: Mild pericardial effusion GA 28 + 6 weeks		Cryptorchidism (U7)	34 + 1
14	Overall inconspicuous development	18	Yes		Psychosocial anomalies: dyslexia, emotional instability, obesity (U11) suspicious EEG findings without epileptic seizures (U11)	39 + 2
15	Overall inconspicuous development	16	Yes		Psychosocial anomalies, reduced learning ability (U10–11)	38 + 5
16	Overall inconspicuous development	9	Yes		Logopedic anomalies: sigmatism (U8-9) Motoric anomalies: muscular hypotension (U8)	37 + 6
17	Overall inconspicuous development	9	Yes		Logopedic anomalies: dyslalia (sibilant sounds), obstructive tubal dysfunction (U10)	41 + 1
18	Overall inconspicuous development	9	Yes		Logopedic anomalies: dyslalia (ch-sounds, U9) Motoric anomalies: asymmetric posture (U10-11)	37 + 6
19	Overall inconspicuous development	3	Yes		Motoric anomalies: mild muscular hypotension, over extensibility of joints (U7a)	40 + 5

*GA* gestational age

^a^Age of the child categorized by period of standardized pediatric examination *(U1-11)* according to German pediatric guidelines (GBA)

*U1-2*: Perinatal. 0–14 days; *U 3–6*: Infant. 15 days–12 months; *U 7–9:* Toddler. 1–5 years; *U 10–11*: Primary school child. 6–9 years

^b^Results from whole genome oligonucleotide array comparative genomic hybridization (aCGH) analysis: Duplication on 4q31; heterozygous deletion on 6p12; and an increase of 350 kb in 19q13

^c^Results from whole genome aCGH analysis: duplication on 4p15

More than half of the children were found to have some anomalies of varying severity in the routine examinations, specialist examinations, and clinical reports, as well as in the parents' perception of the child's health status. From 27 children 13 had minor or major anomalies, another 6 showed an overall inconspicuous development with mild logopedic, psychosocial or motoric impairment, and further 8 children had no anomalies in their development.

Major anomalies (congenital, non-acquired disorders, resulting in permanent severe functional impairment) were diagnosed in two children (7.4%); one had an autism spectrum disorder with ataxia, the other had a global developmental delay leading to severe disability. In the latter case postnatal diagnostics did not reveal a specific diagnosis, but in the whole genome oligonucleotide array comparative genomic hybridization (aCGH) analysis at 2.5 years anomalies with unclear clinical relevance were found. The prenatal karyotype from chorionic villus sampling had been inconspicuous.

Minor anomalies during the development (leading to mild functional impairment while parents experienced the children’s development as normal) were diagnosed in 12 children (44.4%). Recurrent skin edema was reported in two of these children.

Additional mild temporary anomalies were heterogenic, and classified as logopedic (*n* = 8), psychosocial (*n* = 6) and motoric (*n* = 4). For details see Table 3.

### Non-isolated NIHF and results from genetic testing

The most prevalent anomalies of non-isolated NIHF (*n* = 630) were chromosomal (*n* = 456, 64.9%), cardiovascular (*n* = 64, 9.1%), infectious (*n* = 32, 4.6%) and syndromic (*n* = 20, 2.8%). We classified 20 cases with anomalies that did not meet the criteria for one of the diagnostic groups as miscellaneous (2.8%) e.g., cerebral malformations.

All groups of anomalies are listed in Table 4 and details of the classification of each anomaly can be found in the supplementary material in Table S5.

**Table 4:** Classification of the study collective—our data compared to systematic reviews and recent original studies

Group of anomalies	This study 2022	Review: Bellini 2015 [33]	Review: Bellini 2009 [3]	Clinical guideline: SMFM 2015 [1]	Meng 2019 [34]	Laterre 2018 [15]	Moreno 2013 [35]
Isolated/idiopathic %	10.0 (*n* = 70)	19.8	17.8	15–25	28.0	13.7	13.2
Chromosomal %	65.1 (*n* = 456)	9	13.4	7–16	19.8	32.4	28.3
Cardiovascular %	9.1 (*n* = 64)	20.1	21.7	17–35	4.1	9.8	7.5
Infectious %	4.6 (*n* = 32)	7	6.7	5–7	2.6	7.8	7.5
Syndromes %	2.9 (*n* = 20)	5.5	4.4	3–4	0.2	9.8	18.9
Lymphatic %	1.6 (n = 11)	15.0	5.7	5–6	7.8	13.7	5.7
Thoracic %	1.1 (*n* = 8)	2.3	6.0	6	1.7	2	5.7
Urinary tract %	1.0 (*n* = 7)	0.9	2.3	2–3	2.9	1	1.9
Feto-maternal unit %	0.4 (*n* = 3)	4.1	5.6	3–10	3.0	1	3.8
Gastrointestinal %	0.4 (*n* = 3)	1.3	0.5	0.5–4	0.7	0	0
Metabolic %	0.3 (*n* = 2)	1.3	1.1	1–2	–	0	5.7
Extrathoracic tumor %	0.3 (*n* = 2)	0.7	0.7	2–3	–	1	0
Hematologic %	0.3 (*n* = 2)	9.3	10.4	4–12	28.4	7.8	0
Miscellaneous %	2.9 (*n* = 20)	3.6	3.7	3–15	0.8	0	1.9
**Cases (No)**	**700**	**1338**	**5437**		**1004**	**108**	**53**

The karyotype was present in 671 of the total 700 NIHF cases (95.9%). All cases with additional results from molecular genetic testing are listed in the supplementary material in Table S6.

## Discussion

This is the first study to report a large series of 54 spontaneously resolved iNIHF cases with a survival rate of 98.1%. Our observation is consistent with the results of recent studies describing a generally favorable prognosis of prenatally resolved hydrops fetalis [11, 16]. In the univariate analysis by Derderian et al., resolution of hydrops prior to delivery portended better survival rates regardless of the NIHF etiology. They observed resolution in 41 NIHF cases and 76% of these fetuses survived [16]. Gilby et al. reported antenatal resolution in 17 fetuses, six of them with an idiopathic etiology, and all these fetuses survived [11]. The isolated cases within the idiopathic group are not reported in any of these studies. The isolated subgroup may tend to have a better survival prognosis compared to the entire idiopathic group, which includes cases with unclear fetal structural abnormalities and unexplained suspected diagnoses.

Regarding the data from our long-term follow-up, more than half of the children were found to have anomalies of varying severity. From 27 children 13 had minor or major anomalies, another 6 had mild logopedic, psychosocial or motoric impairment. We suspect that even in the event of complete prenatal resolution, the finding of an iNIHF could be an early indication of abnormal child development. However, major developmental anomalies were diagnosed in only two children.

Haverkamp et al. previously reported increased rates of neurological impairment after NIHF and assumed that the neurological long-term outcome depends on the underlying cause of NIHF [27]. They concluded that in particular, survivors with transient benign intrauterine conditions such as idiopathic and lymphatic etiology are at no additional risk for their psychomotor development [27]. This thesis cannot be verified by the results from our long-term follow-up.

Investigating the long-term outcome, we also searched for indications on prenatally undiagnosed conditions that might have been related to the development and resolution of hydrops fetalis. The reports of recurrent skin edema in two children could be indicative of congenital lymphedema. In one of them, we could obtain the results of a postnatal whole genome aCGH analysis. A heterozygote duplication on chromosome 4 was found with unclear clinical significance and unclear correlation to congenital lymphedema. Lately NIHF has been associated to several hereditary lymphedema syndromes; in a systematic review Quinn et al. listed six monogenetic lymphatic diseases that are reported more than once in correlation with NIHF [9].

### Clinical and research implications

In the current American guideline by the Society for Maternal–Fetal Medicine (SMFM), NIHF cases are divided into three prognostic categories: first, cases amenable to fetal therapy; second, those with a fatal prognosis; and third, idiopathic cases with a poor but uncertain prognosis [1]. Regarding the third category, based on our data on the isolated subgroup, we would like to point out that survival rates of iNIHF can be favorable, especially if the NIHF completely resolves prenatally.

We cannot answer whether in these survivors, complete prenatal resolution has beneficial effects on the long-term health. A representative comparative group with prenatal persistence of NIHF is lacking in our long-term data: the only case with persistent mild effusion in the last sonographic control at 29 weeks' gestation had a comparable long-term outcome to cases that were completely resolved prenatally.

To identify iNIHF we recommend at least two ultrasound scans, one of them performed after the first trimester, echocardiography, chromosomal and infectious testing, as well as clarifying prenatal diagnostics in case of any suspected disease.

The extent of other reasonable diagnostics for unexplained NIHF has been increasingly discussed lately: in our study period, screening for metabolic and other monogenetic diseases was not performed regularly, but these conditions are now gaining importance in the diagnostic procedure of unexplained NIHF [2, 9, 36]. In the current SMFM guideline, lysosomal enzyme testing is advised in structurally normal fetuses if available [1].

Recently discussed genetic diagnostics in NIHF include chromosomal microarray analysis (CMA) and whole exome sequencing (WES).

Assuming we had used prenatal CMA for the two cases with severe functional impairment in our study, we hypothesize that in at least one of them (Case 2 in Table 3), this may have prenatally revealed the genetic abnormalities that were found in postnatal CMA. Possibly the anomaly could have been considered in prenatal counselling. However, the clinical impact of this postnatally diagnosed genetic variant is still unknown. Furthermore, current studies suggest that CMA in general has a low diagnostic utility for NIHF [2, 37].

We cannot estimate how many of our cases would have benefited from prenatal WES which was not routinely used in clinical testing in our early study period or even nowadays [38]. Using WES Sparks et al. were able to identify a pathogenic or likely pathogenic variant in 29% of NIHF cases unexplained by standard genetic testing [2]. In a meta-analysis of Mone et al. the pooled incremental yield of prenatal exome sequencing over chromosomal microarray analysis or karyotyping was 21% in iNIHF [5].

Monogenetic disorders are increasingly understood as one of the major contributing etiologies to NIHF [2, 5, 8, 9]. Using WES further progress in terms of understanding the pathogenesis and resolution of NIHF can be expected. Simultaneously, it should be borne in mind that there are still ethical aspects to be considered when using WES e.g., regarding the diagnosis of secondary genetic results such as predisposition to cancer or cardiovascular disease [2].

### Strengths and limitations

One strength of our study is the large number of total NIHF cases which allowed the identification of a representative isolated subgroup. The distinct definition of iNIHF minimized uncontrolled variability within this group.

Another strength of our study is that we achieved a comprehensive long-term follow-up with a mean of 10.9 years for 27 survivors of iNIHF. Patient informed consent, based on detailed study information sent and explained to the parents and children enabled us to obtain multidisciplinary long-term data. We consider the records of the children's health status based on parental perception to be particularly valuable for the prenatal counselling situation.

There are certain limitations of this study due to the retrospective study design and large study period of 19 years. This limited the availability of postnatal outcomes: from all reviewed NIHF cases 5.2% had to be excluded due to missing outcome information; for the long-term outcome of the iNIHF survivors we were able to obtain pediatric reports from only 47.4%.

Assuming that parents are more likely to participate when development is perceived as normal [39], the number of abnormal developments might be underestimated. Therefore, we cannot predict the long-term health of all fetuses with iNIHF. However, the outcome reports provide data on the spectrum of possible anomalies and functional limitations after prenatal iNIHF.

We investigated whether the exclusion criteria in our study led to a non-response bias concerning the iNIHF survival rates. We estimated the maximal possible error that could have been caused by this bias: from the cases excluded due to a lack of postnatal information, three fetuses may have been isolated as they did not show any anomalies in the complete prenatal diagnostic workup. Assuming all of them showed a spontaneous resolution and had a fatal outcome, the baby-take-home rate for the iNIHF group with prenatal resolution would decrease from 98.1 to 93.0%.

Furthermore, our study provides data only on the outcome of fetuses that survived to the second trimester, since we required a second-trimester ultrasound examination that did not reveal phenotypic anomalies other than hydrops fetalis to classify a case as iNIHF. Therefore, no predictions on the survival prognosis of NIHF at the time of a first-trimester examination can be made from our data. Survival rates could be significantly lower if a first-trimester ultrasound without other anomalies in addition to hydrops fetalis was considered sufficient for the diagnosis of iNIHF.

Other limitations of our study collective imply a preselection bias since the study was conducted in a prenatal referral center. Pregnant women from a large population are referred in case of abnormalities or irregular development. We were not able to draw conclusions about incidences in the general population, but this did not prevent the clear identification of iNIHF within the study population.

## Conclusion

In our collective, the most common course of iNIHF was spontaneous resolution. Clinicians should thus be aware that the prognosis of early diagnosed NIHF may be favorable if the hydrops remains the only pathological finding. Controlled studies in a general population need to be performed to verify whether this should be included in clinical guidelines for counselling pregnancies with NIHF. Our data on the long-term outcomes are consistent with the assumption of an increased rate of functional impairments after prenatally resolved iNIHF. Recurrent skin edema in the pediatric development might be indicative of prenatally undiagnosed congenital lymphedema.

## Supplementary Information

Below is the link to the electronic supplementary material.

Supplementary file1 (PDF 224 KB)
