# Supplementary material for: Isolated non-immune hydrops fetalis: an observational study on complete spontaneous resolution, perinatal outcome, and long-term follow-up
Source: Arch Gynecol Obstet. 2022 Aug 22;308(2):487–97. doi: 10.1007/s00404-022-06731-w (PMC9395874; doi:10.1007/s00404-022-06731-w)
Supplement: Supplementary file 1 — Supplementary file1 (PDF 224 KB) [file 404_2022_6731_MOESM1_ESM.pdf]

Appendix 1: Classification of the study collective – Details of prenatal ultrasound and laboratory findings

Group and details of anomalies	No	Group and details of anomalies	No
**Isolated**	**70**	**Lymphatic**	**11**
**Chromosomal**	**456**	Cystic hygroma colli	7
Trisomy 21	173	Chylothorax	3
Turner	127	Lymphatic dysplasia	1
Trisomy 18	106	**Thoracic**	**8**
Trisomy 13	21	Diaphragmatic hernia	3
Unbalanced structural anomaly	12	CCAML	3
Triploid	9	Ectopia cordis	1
Trisomy 14	2	CHAOS	1
Tetraploid	2	**Urogenital**	**7**
Trisomy 22	1	Potter	1
Tetrasomy 12p	1	Obstructive Uropathy	1
Trisomy 23	1	Infravesical obstruction	1
Trisomy 16	1	Renal agenesis	1
**Cardiovascular**	**64**	Renal vein thrombosis	1
Cardiac arrhythmia	12	Renal dysfunction	1
Other heart defect	8	LUTO	1
Valvular heart disease	7	**Feto-maternal unit**	**3**
Hypoplastic left heart	6	Placental hematoma	1
Cardiomyopathy	5	Villous maturation disorder	1
VSD	5	Umbilical hernia	1
Arteriovenous fistula	3	**Gastrointestinal**	**3**
AVSD	3	Intrahepatic venous anomaly	1
Fallot	2	Omphalocele	1
Ebstein-Anomalia	2	Lesions of liver parenchyma	1
Coarctation of the aorta	2	**Metabolic**	**2**
Univentricular heart defect	2	Thanatophoric dwarfism	1
Interrupted aortic arch	2	Mucopolysaccharidosis	1
Hypoplastic right heart	2	**Extrathoracic tumor**	**2**
Double outlet right ventricle	1	Teratoma	2
ASD	1	**Hematologic**	**2**
Agenesis of ductus venosus	1	Non-immune erythroblastosis	1
**Infection**	**32**	Unclear anemia	1
Parvovirus	25	**Miscellaneous**	**20**
CMV	5	Unspecific syndromic	8
Toxoplasmosis	1	Asphyxiated skeletal dysplasia	2
Coxsackie	1	Cerebral malformation	2
**Syndromes**	**20**	Anencephaly-Exencephaly	1
Pena shokeir	4	Exencephaly	1
Pterygium	3	Iniencephaly	1
Arthrogryposis congenita multiplex	2	Cleft lip and palate	1
Cantrell	2	Ventral cleavage	1
Dandy Walker	2	Pachygyria	1
Noonan	2	Gastroschisis	1
Right sided atrial isomerism	1	Rachischisis	1
			**Total 700**

ASD: atrial septal defect; AVSD: atrioventricular septal defect; CCAML: congenital cystic adenomatoid malformation; CHAOS: congenital high airway obstruction syndrome; CMV: Cytomegalovirus; LUTO: lower urinary tract obstruction; VSD: ventricular septal defect4

Appendix 2: Results from prenatal and postnatal molecular biology

Case	Group	Prenatal classification from ultrasound and laboratory findings	Karyotype	Techniques of molecular biology	Suspected disease	Genloci/a-cGH Results	Conspicuous findings
**1[a]**	**Isolated NIHF**	Isolated NIHF	46,XY	a-CGH (prenatal)	Chromosomal microdeletions and microduplications	No abnormal a-CGH Results	No
**2**	Non-isolated NIHF	Unbalanced structural anomaly	46,XXadd(4)(p15.2)	a-CGH (prenatal)	Chromosomal microdeletions and microduplications	1. Deletion 4p16.3 to 4p16.1 (size 8.8Mb, containing 94 genes).<br>2. Duplication 4p16.1 to 4p15.31 (size 10 Mb, containing 29 genes)<br>3. Duplication 4q25 to 28.3 (size 24.7 Mb, 90 genes)<br>4. Duplication 4q28.3 to q35.2 (size 57.6 Mb, 184 genes)	**Yes**
**3**	Non-isolated NIHF	Cardiac arrhythmia	46,XY	Direct DNA sequencing (prenatal)	Noonan-Syndrome. Genes: PtPn11, SOS1, RAF1 and KRAS	PTPN11: exons 1-15. SOS1 exons: 3,4,6,7,10,11,13,14,15. RAF1: exons 7, 14, 17. Kras: 2,3,4,5,6.	No
**4**	Non-isolated NIHF	Hygroma colli	46,XX	Direct DNA sequencing (prenatal)	Noonan-Syndrome. Genes: PtPn11, SOS1, RAF1 and KRAS	PTPN11: exons 1-15. SOS1 exons: 3,4,6,7,10,11,13,14,15. RAF1: exons 7, 14, 17. Kras: 2,3,4,5,6.	No
**5**	Non-isolated NIHF	CMV Infection	46, XY	DNA sequencing with multiplex PCR and oligo-ligation (prenatal)	Cystic fibrosis. Gen: CTFR.	31 mutations	No
**6**	Non-isolated NIHF	Ventricular septal defect	46, XY	DNA sequencing with multiplex PCR and oligo-ligation (prenatal)	Noonan-Syndrome. Gen: PtPn11	PTPN11: exons 3,8,13	No
**7**	Non-isolated NIHF	Ebstein-Anomalia	46,XX	MLPA (Multiplex ligation-dependent probe amplification) (prenatal)	1. Beckwith-Wiedemann-Syndrome<br>2. Noonan-Syndrome	Quantitative analysis: Exclusion of deletions or duplications on 11p15; Microsatellite analysis: Exclusion of uniparental disomy 11 (No CDKN1C sequencing); Methylation analysis: KvDMR1 und H19-DMR	No
**8**	Non-isolated NIHF	Hypoplastic left heart	46,XX	Molecular cytogenetic FisH (prenatal)	DiGeorge-Syndrome. Gen: 22q11.2	D22S553, D22S609, D22S942	No
**9**	Non-isolated NIHF	Tetralogy of Fallot	46,XX	Molecular cytogenetic FisH (prenatal)	DiGeorge-Syndrome. Gen: 22q11.2	D22S553, D22S609, D22S942	No
**10**	Non-isolated NIHF	Complex structural heart defect	46,XY	Molecular cytogenetic FisH (prenatal)	1. DiGeorge 22q11<br>2. Noonan-Syndrome 10p14	22q11: D22S553, D22S609, D22S942.	No
**11[b]**	**Isolated NIHF**	Isolated NIHF	46,XX	a-CGH **(postnatal)**	Unspecified syndrome	1. Duplication 4q31<br>2. Heterozygous deletion 6p12<br>3. Insertion 19q13 (size 350 kb)	**Yes**
**12[c]**	**Isolated NIHF**	Isolated NIHF	46,XY	a-CGH **(postnatal)**	1. Hellkamp-Syndrome<br>2. Noonan-Syndrome<br>3. Metabolic disorder	Duplication 4p15.33 (size 472-495 kb)	**Yes**

aCGH: array comparative genomic hybridization; NIHF: non-immune hydrops fetalis

[a] Case 12 in the individual case survey on anomalies in the long-term development of iNIHF (Table 3)

[b] Case 2 in the individual case survey on anomalies in the long-term development of iNIHF (Table 3)

[c] Case 8 in the individual case survey on anomalies in the long-term development of iNIHF (Table 3)
